# Hand–mobile phone culture-burden associations and taxon-level concordance among healthcare-associated personnel: a paired cross-sectional study

**DOI:** 10.3389/fcimb.2026.1929858

**Published:** 2026-08-26

**Authors:** Wenjing Jiang, Chunli Song, Li Cao, Yuhan Zhang, Xiaoyu Liao, Yu Chen, Xianyu You, Suchuan Zhang, Juan Tang

**Affiliations:** 1 Department of Hospital Infection Management, Zigong First People’s Hospital, Zigong, China; 2 West China School of Public Health, Sichuan University, Chengdu, China; 3 Department of Clinical Laboratory, Zigong First People’s Hospital, Zigong, China; 4 Department of Infectious Diseases, Zigong First People’s Hospital, Zigong, China

**Keywords:** hand hygiene, healthcare-associated personnel, infection prevention, microbial contamination, mobile phones, paired sampling, taxon-level concordance

## Abstract

**Background:**

Hands and mobile phones are frequently handled during healthcare delivery and caregiving, but their participant-level culture-burden association and taxonomic overlap remain incompletely characterized. We examined whether paired hand and mobile-phone contamination was associated within individuals and whether the two sites showed overlap or discordant detection among culture-defined taxa.

**Methods:**

We conducted a paired cross-sectional study in a tertiary Grade A general hospital between April and June 2026. Physicians, nurses, and professional caregivers each provided one hand sample and one paired mobile-phone sample. Because hand and mobile-phone burdens were expressed in different site-specific units, absolute burdens were not directly compared. Spearman rank correlation was the primary association analysis; separately standardized log-burden regression models were used for covariate adjustment. Binary culture-defined taxon profiles were evaluated using overlap metrics, exploratory paired-label multivariate analyses, and exact McNemar tests.

**Results:**

A total of 126 participants were included. The median culture burden was 11.67 CFU/cm² (IQR, 1.67–54.17) for hands and 950.00 CFU/phone (IQR, 300.00–2575.00) for mobile phones. The site-specific burdens were positively associated (Spearman rho=0.514, P<0.001). After separate standardization of log10(CFU + 1) values within each site, the association remained in the core adjusted model (standardized beta=0.512, 95% CI 0.291–0.732; P<0.001) and the extended sensitivity model (standardized beta=0.497, 95% CI 0.276–0.718; P<0.001). Median Jaccard similarity was 0.50 among 119 pairs with at least one detected non-Other taxon, and the median shared-taxon count was 1 across all 126 pairs. Exploratory multivariate findings were sensitive to the analysis set and were not reproduced when restricted to pairs with non-Other taxa detected at both sites. Coagulase-negative staphylococci were the only prespecified taxon with significant discordant detection after multiplicity correction.

**Conclusions:**

Paired hand and mobile-phone culture burdens showed a positive participant-level association and partial culture-defined taxonomic overlap. The cross-sectional culture-based design does not establish direct microbial transmission or causality. Prospective studies should evaluate whether coordinated hand-hygiene and device-cleaning approaches improve microbiological outcomes.

## 1 Introduction

Microbial transmission in healthcare settings remains a major concern for infection prevention and control (Haque et al., 2018; World Health Organization, 2022). The World Health Organization has emphasized that health care-associated infections continue to occur in acute-care hospitals and that hand hygiene is a cornerstone intervention for preventing avoidable infections; nevertheless, compliance with hand hygiene recommendations remains suboptimal in real-world care settings (Allegranzi and Pittet, 2009; Pittet et al., 2009). Beyond direct patient contact, contaminated high-touch surfaces may contribute to the persistence and transfer of microorganisms within clinical environments (Kramer et al., 2006; Otter et al., 2011; Dancer, 2014). With the widespread use of mobile phones during clinical work and caregiving activities, personal mobile devices represent a distinct category of high-touch personal surfaces: they are repeatedly handled, carried across work areas, and exposed to hands, clothing, patient surroundings, and shared surfaces, yet they are often not subjected to the same standardized cleaning and disinfection practices as medical equipment (Huslage et al., 2010; Weber et al., 2010; Donskey, 2013).

Previous studies have shown that mobile phones used by healthcare workers are frequently contaminated with skin-associated organisms, environmental organisms, and occasional potential pathogens (Brady et al., 2006; Goldblatt et al., 2007; Ulger et al., 2009). Systematic reviews indicate that mobile-phone contamination is common across countries and care settings, although direct links to clinical infection remain unproven (Olsen et al., 2020; De Groote et al., 2022; Zenbaba et al., 2023). An earlier literature review likewise documented frequent contamination and inconsistent decontamination practices (Ulger et al., 2015). Recent culture-based work has similarly recovered potentially pathogenic bacteria from phones used by medical students and healthcare workers (Altunisik Toplu et al., 2026). Studies sampling both hands and phones have reported organisms at both sites, but culture-level similarity alone cannot establish a common source or direction of transfer (Jeske et al., 2007; Yao et al., 2022). Mobile phones may therefore represent related high-touch surfaces within the same participant-level contact environment as hands.

Important evidence gaps remain. Many studies have focused on contamination prevalence, colony counts, dominant isolates, or behavioral correlates at a single sampling site (Olsen et al., 2020; De Groote et al., 2022; Zenbaba et al., 2023). Even when hand and mobile-phone samples were collected from the same individuals, analyses have often emphasized positivity or apparently similar isolates rather than participant-level association between site-specific culture burdens. Conventional surveys also provide limited information about the overlap of culture-defined taxa or taxon-specific discordant detection. A paired approach can address these questions while preserving the distinct sampling units of hands and phones.

In this paired cross-sectional study, we collected matched hand and mobile-phone samples from physicians, nurses, and professional caregivers in a tertiary general hospital. Healthcare-associated personnel were defined as these three groups because each provided direct care services within the hospital. We assessed participant-level associations between site-specific CFU burdens, culture-defined taxon overlap, and taxon-specific discordant detection. Clinically relevant antimicrobial-resistant isolates were summarized descriptively by participant role, department, and sampling site.

## 2 Materials and methods

### 2.1 Study design and participants

This paired cross-sectional study was conducted between April and June 2026 in a tertiary Grade A general hospital. Physicians, nurses, and professional caregivers were recruited from clinical departments using convenience sampling.

The study was designed as a within-participant paired sampling investigation, with one hand sample and one mobile-phone sample collected from each participant during the same sampling window.

Eligible participants were adults aged ≥18 years working or providing professional caregiving services in the hospital who agreed to complete the questionnaire and microbiological sampling and provided both paired samples. Participants were excluded if paired sampling was incomplete, sample labels were mismatched, or key questionnaire or microbiological data were missing.

A total of 126 participants were included in the final analysis, each contributing paired hand and mobile-phone samples. The study was approved by the Ethics Committee of Zigong First People’s Hospital (No. 2026089). All participants were informed of the study purpose and procedures and provided informed consent before questionnaire completion and sample collection.

### 2.2 Questionnaire and covariates

A structured questionnaire was used to collect demographic characteristics, occupational information, hand hygiene behaviors, and mobile-phone use and cleaning practices. The questionnaire was administered online via Wenjuanxing, with trained staff assisting participants when needed.

Variables included age, sex, occupation, department, daily handwashing frequency, daily alcohol-based handrub use, mobile-phone use duration, and mobile-phone cleaning frequency. Age, daily handwashing, daily alcohol-based handrub use, and mobile-phone use duration were modeled as numeric variables; sex, occupation, and department were categorical variables; and mobile-phone cleaning frequency was entered as the recorded four-level ordinal score. Category midpoints were used for interval responses. Covariates were selected *a priori* based on their potential associations with hand contamination, mobile-phone contamination, or both. The core adjusted model included age, sex, occupation, department, daily handwashing frequency, daily alcohol-based handrub use, and mobile-phone cleaning frequency; mobile-phone use duration was added in the extended sensitivity model.

### 2.3 Materials and laboratory instruments

Sterile neutralizing solution (10 mL per tube), sterile swabs, and 90-mm blood agar plates were obtained from Chengdu Ruiqi Medical Technology Co., Ltd. The same sterile neutralizing solution was used for swab moistening, sample transport, and microbiological processing of both hand and mobile-phone samples. Microbial identification was performed using the VITEK MS system (BioMérieux, France) according to the manufacturer’s instructions. Antimicrobial susceptibility testing was performed using the VITEK 2 Compact system with AST-GP67 and AST-N335 cards for Gram-positive and Gram-negative organisms, respectively, and interpreted according to CLSI M100, 35th edition (2025) (Clinical and Laboratory Standards Institute, 2025).

### 2.4 Sampling procedures

Hand and mobile-phone sampling followed the Hygienic Standard for Disinfection in Hospitals and was performed by trained personnel under aseptic conditions (General Administration of Quality Supervision, Inspection and Quarantine of the People's Republic of China and Standardization Administration of China, 2012). The hand-sampling procedure followed the medical-staff hand hygiene sampling standard, and the corresponding conversion factor used for CFU calculation was 100.

For hand sampling, a sterile swab moistened with sterile neutralizing solution was used to sample both hands. The curved finger surfaces were swabbed from the base to the fingertips and back twice on each hand, covering an estimated surface area of 30 cm² per hand, while rotating the swab and avoiding direct contact with unsampled areas. The swab was then placed into a tube containing 10 mL sterile neutralizing solution for transport.

For mobile-phone sampling, one sterile swab moistened with sterile neutralizing solution was used to swab the front and back surfaces of the routinely used mobile phone once with full-coverage back-and-forth strokes while rotating the swab in the same manner as for hand sampling. When present, the external surface of the phone case was included. Sampling was conducted during normal work shifts. Participants were not sampled while actively providing patient care, performing procedures, or handling contaminated items; they were not wearing gloves; hand hygiene was not performed immediately before sampling; and mobile phones were not wiped, disinfected, or fitted with a new phone case immediately before sampling. Trained samplers confirmed a relatively stable routine work state before collection. Exact intervals since the most recent hand hygiene episode, phone use, patient contact, or phone cleaning were not recorded.

### 2.5 Microbiological processing

After mixing, 100 µL of sample suspension was inoculated onto one 90-mm blood agar plate per sample and spread evenly using a disposable sterile inoculating loop. Plates were incubated at 36 ± 1°C for 48 hours. Colony-forming units (CFU) were counted, and samples without visible growth were recorded as 0 CFU. CFU values were converted using site-specific formulas. For hand samples, hand CFU burden was calculated as: hand CFU/cm² = colony count on the plate × conversion factor/(30 cm² × 2), where the conversion factor was 100 and the denominator represented the sampled area of both hands. For mobile-phone samples, mobile-phone CFU burden was calculated as: mobile-phone CFU/phone = colony count on the plate × conversion factor, where the conversion factor was 100 based on the 10-mL sampling solution and 100-µL inoculation volume.

Isolates were identified to the species or genus level where possible using VITEK MS. Organisms with limited resolution or sparse detection were grouped into predefined culture-defined taxa, including coagulase-negative staphylococci, *Corynebacterium* spp., *Micrococcus* spp., Enterobacterales, non-fermenting Gram-negative bacilli, and residual descriptive categories. These groups were analyzed as culture-defined taxa rather than strict species. Antimicrobial susceptibility testing was performed for clinically relevant *S. aureus* and Gram-negative isolates requiring resistance interpretation. MRSA was defined as *S. aureus* classified as cefoxitin- or oxacillin-resistant, and CRAB as *A. baumannii* classified as resistant to imipenem or meropenem, according to CLSI M100, 35th edition (2025). Each resistant recovery was counted as one isolate-site record; a participant could contribute records from both paired sites. Resistant isolates were summarized descriptively by department, participant role, and sampling site.

### 2.6 Data processing

The participant was the primary unit of analysis, with paired hand and mobile-phone samples treated as within-participant observations. Microbiological data were cleaned and standardized before analysis. Taxon-level detection was defined at the participant-site level and converted into binary presence/absence matrices. Concordance and discordance analyses were therefore based on paired participant-level matrices of culture-defined taxa rather than raw detection counts.

### 2.7 Statistical analysis

All analyses were performed using Python 3.12.13, pandas 3.0.1, and NumPy 2.3.5 with custom scripts (McKinney, 2010; Harris et al., 2020). The participant was the unit of analysis, with one paired hand and mobile-phone sample per participant. Continuous variables were summarized as medians and interquartile ranges (IQRs), and categorical variables as counts and percentages.

CFU burden was summarized by sampling site using site-specific CFU burden metrics and log10(CFU + 1)-transformed values. Occupational differences in CFU burden were assessed separately for hand and mobile-phone samples using Kruskal-Wallis tests, with Dunn-Bonferroni *post-hoc* comparisons when appropriate; these occupational analyses were reported as supplementary analyses.

Hand and mobile-phone burdens were expressed in site-specific units (CFU/cm² and CFU/phone, respectively), so their absolute values were summarized separately. Log10(CFU + 1) transformation was applied to reduce right skewness. Spearman rank correlation was the primary participant-level association analysis. For covariate-adjusted analyses, the hand and mobile-phone log-burden variables were standardized separately to z scores using their full-sample means and standard deviations. A standardized beta therefore represents the standard-deviation change in mobile-phone log burden associated with a one-standard-deviation increase in hand log burden. The core model included age, sex, occupation, department, daily handwashing frequency, daily alcohol-based handrub use, and mobile-phone cleaning frequency; mobile-phone use duration was added in the extended sensitivity model. Complete-case analysis was used, and no model covariates were missing. HC3 robust standard errors were used (MacKinnon and White, 1985). Variance inflation factors, leverage, Cook’s distance, and studentized residuals were examined. Models were refitted after excluding observations with Cook’s distance >4/n or leverage >2p/n, retaining the full-sample standardization parameters. Unstandardized log-linear coefficients were reported as secondary analyses.

Taxon presence or absence was defined at the participant-site level, with the residual Other category excluded from overlap and multivariate analyses. Taxon richness and shared-taxon count were summarized for all 126 pairs. Jaccard similarity and binary Bray–Curtis distance were summarized for the 119 pairs with at least one non-Other taxon detected; Jaccard similarity was the number of shared taxa divided by the number detected at either site. Exploratory site-associated distributional differences were assessed in all 126 pairs by paired-label PERMANOVA on binary Bray–Curtis distances using 9,999 within-participant label permutations (random seed 20260705), implemented with custom NumPy routines (Bray and Curtis, 1957; Anderson, 2001). For the multivariate distance matrix, the distance between two all-zero profiles was set to 0 and that between an all-zero and a nonzero profile to 1. A permutation-based, PERMDISP-style sensitivity analysis used the absolute difference in mean distance to site centroids, also with 9,999 within-participant label permutations (random seed 20260706) (Anderson, 2006). The two tests were interpreted jointly. As a *post hoc* sensitivity analysis, both were repeated among the 99 pairs with at least one non-Other taxon detected at each site.

Taxon-specific discordance was evaluated using exact McNemar tests on participant-level paired 2×2 presence/absence tables (McNemar, 1947). Six prespecified, interpretable taxa comprised one fixed testing family; the same six tests were used for both Bonferroni and Benjamini–Hochberg false-discovery-rate adjustment (Benjamini and Hochberg, 1995). The residual Other category was retained only in descriptive summaries. Resistant isolates were summarized descriptively because event counts were low. All tests were two-sided, with P<0.05 considered statistically significant.

## 3 Results

### 3.1 Study population and site-specific CFU burden

A total of 126 participants with paired samples were included. Median age was 37 years (IQR, 32–55), and 88 participants (69.8%) were female. The sample comprised 47 professional caregivers (37.3%), 41 physicians (32.5%), and 38 nurses (30.2%); 58 participants (46.0%) worked in internal medicine, 46 (36.5%) in surgery, and 22 (17.5%) in the ICU. Median culture burden was 11.67 CFU/cm² (IQR, 1.67–54.17) for hands and 950.00 CFU/phone (IQR, 300.00–2575.00) for mobile phones. Corresponding median log10(CFU + 1) values were 1.103 (IQR, 0.427–1.742) and 2.978 (IQR, 2.479–3.411), respectively (**Table 1**). These site-specific values describe different sampling units and were not compared as absolute burden levels.

**Table 1:** Site-specific culture burden and participant-level hand–mobile phone association. [Revised].

Analysis	n	Estimate/summary	95% CI or model fit	P value or zero-CFU, n (%)
Hand culture burden	126	11.67 (1.67-54.17) CFU/cm²	log10(CFU + 1): 1.103 (0.427-1.742)	25 (19.8%)
Mobile-phone culture burden	126	950.00 (300.00-2575.00) CFU/phone	log10(CFU + 1): 2.978 (2.479-3.411)	8 (6.3%)
Spearman rank association	126	rho=0.514	Primary association analysis	<0.001
Unadjusted standardized regression	126	0.503 (0.316 to 0.691)	adjusted R²=0.247	<0.001
Core adjusted standardized regression	126	0.512 (0.291 to 0.732)	adjusted R²=0.217	<0.001
Extended adjusted standardized regression	126	0.497 (0.276 to 0.718)	adjusted R²=0.225	<0.001

Site-specific burden values are median (IQR); zero-CFU values are n (%). Absolute hand (CFU/cm²) and mobile-phone (CFU/phone) burdens were not directly compared. Standardized beta is the SD change in mobile-phone log burden per 1-SD increase in hand log burden. CI, confidence interval.

In site-specific occupational comparisons, hand CFU differed across occupational groups (Kruskal-Wallis H = 11.886, P = 0.003), driven by a Dunn-Bonferroni difference between nurses and professional caregivers (adjusted P = 0.002); mobile-phone CFU did not differ significantly by occupation (H = 4.947, P = 0.082) (**Supplementary Tables 1, S2**).

### 3.2 Participant-level association between hand and mobile-phone culture burdens

**Figure 1:**
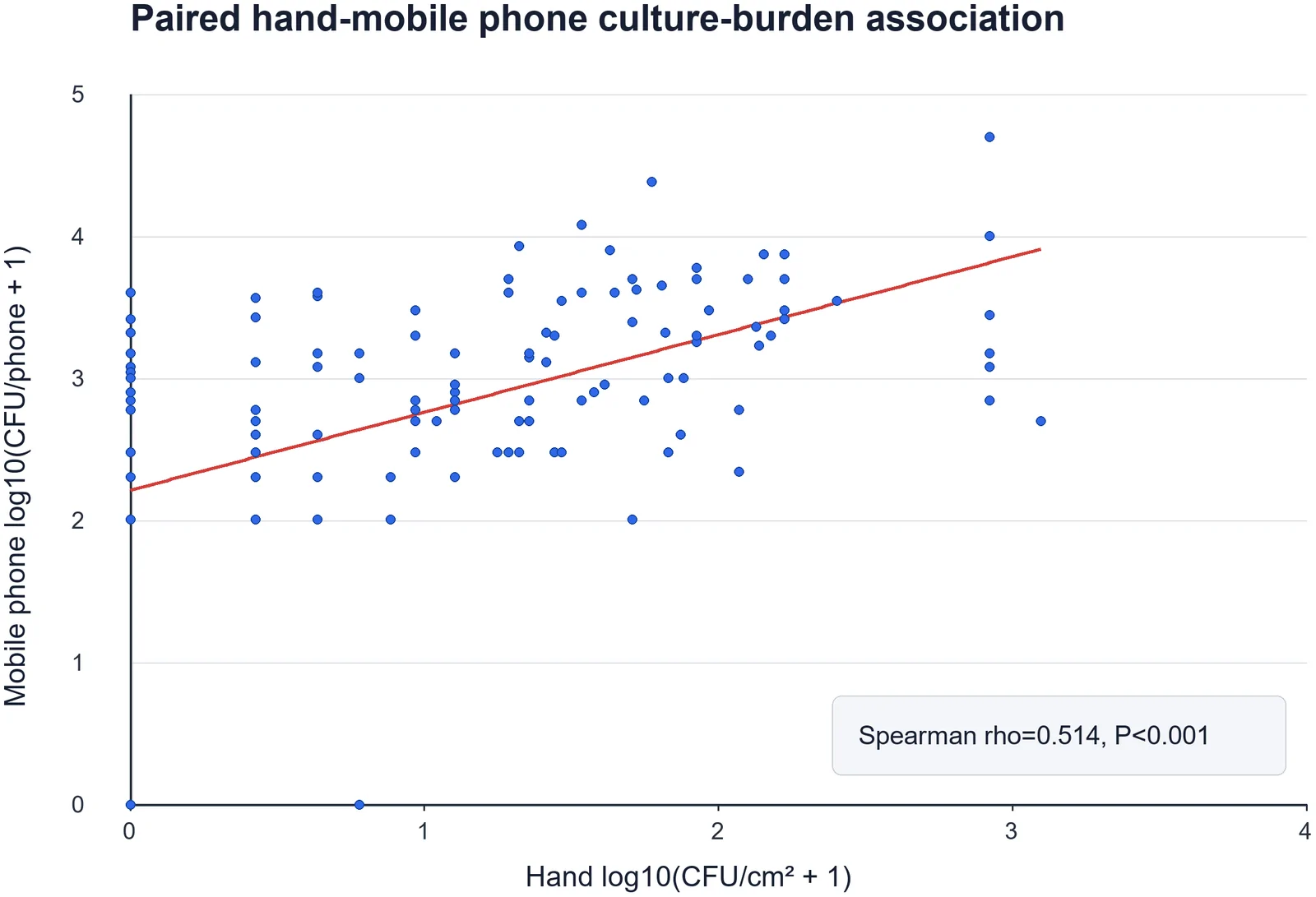
Participant-level association between hand and mobile-phone culture burden. Points show paired site-specific log10(CFU + 1) values. The line is an unadjusted linear fit for visualization; Spearman rho is the primary association statistic. Axis labels retain the distinct hand and mobile-phone units.

Hand and mobile-phone culture burdens showed a positive participant-level rank association (Spearman rho=0.514, P<0.001; **Figure 1**). In models using separately standardized log10(CFU + 1) variables, the unadjusted standardized beta was 0.503 (95% CI 0.316–0.691; P<0.001). The association remained in the core adjusted model (standardized beta=0.512, 95% CI 0.291–0.732; adjusted R²=0.217; P<0.001) and the extended model including mobile-phone use duration (standardized beta=0.497, 95% CI 0.276–0.718; adjusted R²=0.225; P<0.001; **Table 1**). Full standardized coefficients are reported in **Supplementary Table 3**; secondary unstandardized models, influence diagnostics, variance inflation factors, and influence-exclusion analyses are reported in **Supplementary Tables 4–S7**. After exclusion of 12 observations meeting the prespecified influence criteria, the association remained positive but was attenuated (core standardized beta=0.286, 95% CI 0.158–0.413; extended standardized beta=0.266, 95% CI 0.139–0.394; both P<0.001; **Supplementary Table 7**).

### 3.3 Taxon-level overlap and culture-defined taxonomic concordance

**Figure 2:**
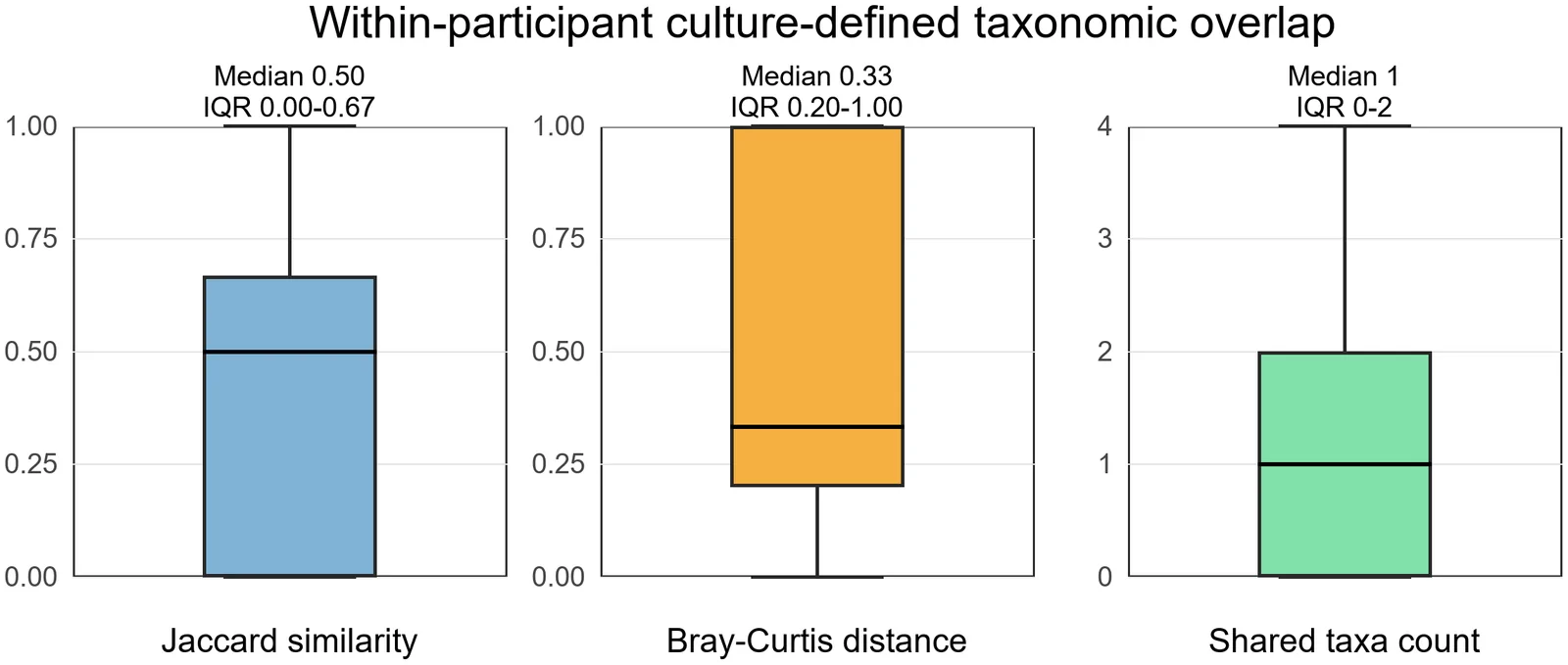
Within-participant culture-defined taxonomic overlap metrics. Box plots summarize Jaccard similarity and Bray–Curtis distance among the 119 pairs with at least one non-Other taxon detected, and shared-taxon count across all 126 paired samples.

Median non-Other taxon richness was 2 in hand samples (IQR, 1–3) and 2 in mobile-phone samples (IQR, 1–3), and the median shared-taxon count across all 126 pairs was 1 (IQR, 0–2) (**Table 2**). Among 119 pairs with at least one non-Other taxon detected, median Jaccard similarity was 0.50 (IQR, 0.00–0.67) and median binary Bray–Curtis distance was 0.333 (IQR, 0.20–1.00) (**Figure 2**; **Table 2**). CoNS, *Corynebacterium* spp., and *Micrococcus* spp. were the most frequently detected taxa at both sites (**Supplementary Table 8**; **Supplementary Figure 1**). In the full sample, exploratory paired-label PERMANOVA was significant (pseudo-F=5.886, P<0.001), as was the dispersion analysis (mean distance to centroid: hand=0.548, mobile phone=0.449; absolute difference=0.099; P<0.001) (**Table 2**; **Supplementary Table 9**). Among the 99 pairs with non-Other taxa detected at both sites, neither PERMANOVA (pseudo-F=1.020, P = 0.271) nor the dispersion analysis (absolute difference=0.043, P = 0.081) was significant. Thus, the full-sample multivariate result was not retained in the both-positive subset.

**Table 2:** Within-participant culture-defined taxonomic overlap and exploratory site analyses. [Revised].

Metric	n	Summary
Hand non-Other taxon richness	126	2.00 (1.00-3.00)
Mobile-phone non-Other taxon richness	126	2.00 (1.00-3.00)
Shared non-Other taxa count	126	1.00 (0.00-2.00)
Jaccard similarity	119	0.50 (0.00-0.67)
Bray–Curtis distance	119	0.33 (0.20-1.00)
Paired-label PERMANOVA	126 pairs (252 site profiles)	pseudo-F=5.886; 9,999-permutation P<0.001
Dispersion sensitivity analysis	126 pairs (252 site profiles)	mean distance: hand=0.548, phone=0.449; absolute difference=0.099; P<0.001

Metrics used binary presence/absence data after exclusion of the residual Other category. Jaccard and paired Bray–Curtis summaries included 119 pairs with at least one non-Other taxon detected. PERMANOVA was exploratory; the dispersion analysis used the absolute difference in mean distance to site centroids. Both tests used 9,999 within-participant label permutations. Results for the 99-pair both-positive sensitivity set are provided in **Supplementary Table 9**.

### 3.4 Taxon-level paired discordance and clinically relevant resistant isolates

**Figure 3:**
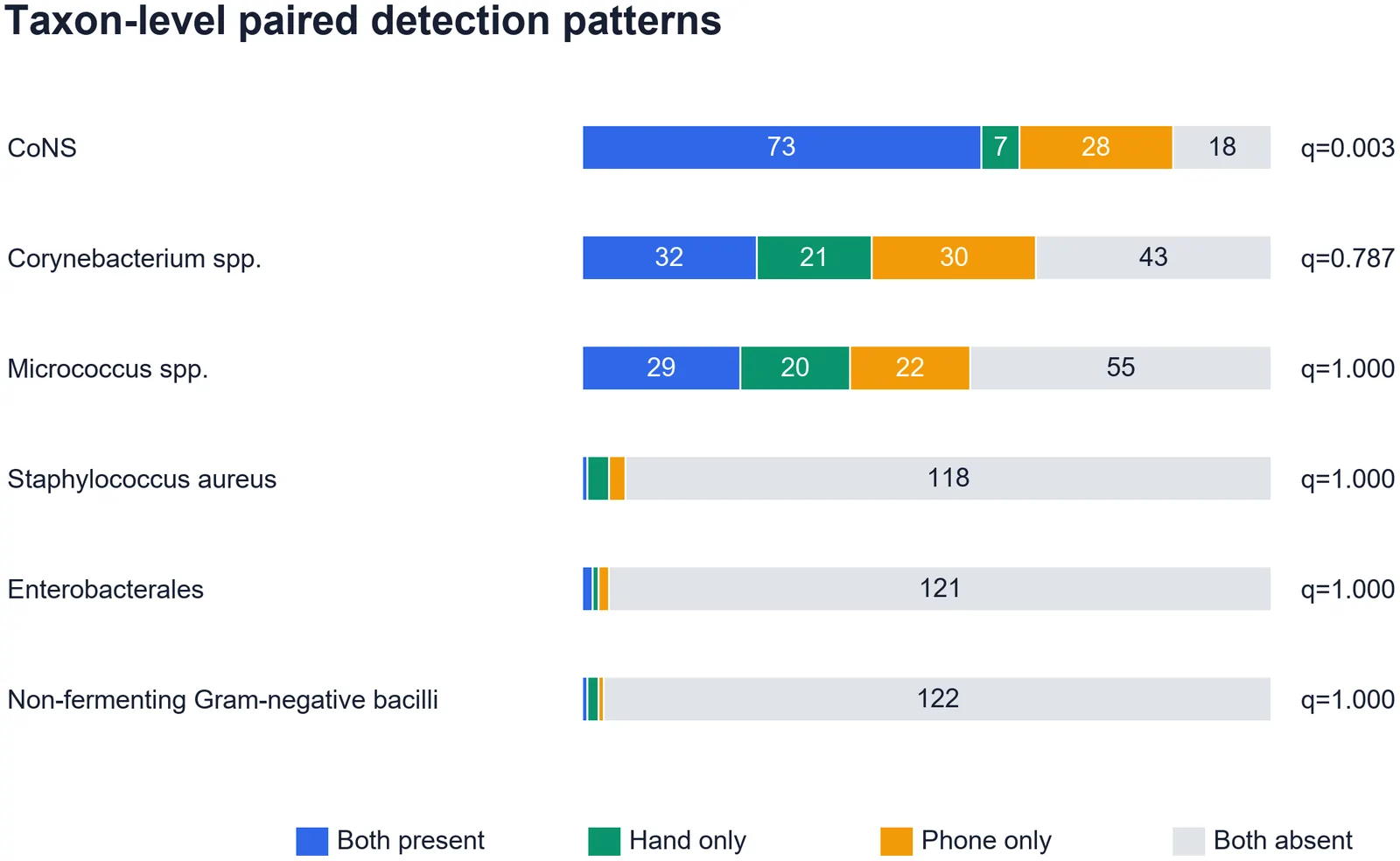
Taxon-level paired detection patterns. Stacked bars show the four paired detection states for the six predefined culture-defined taxa included in the primary McNemar analysis. q values denote Benjamini–Hochberg-adjusted McNemar results across the taxa displayed.

Exact McNemar tests used participant-level paired presence/absence tables for the six prespecified taxa. CoNS was the only taxon remaining significant after multiplicity correction: 73 pairs were positive at both sites, 7 were hand-only, 28 were phone-only, and 18 were negative at both sites (exact P<0.001; FDR q=0.003; Bonferroni-adjusted P = 0.003; **Table 3**; **Figure 3**). Six antimicrobial-resistant isolates were recovered from six participant-site samples contributed by five participants, comprising three MRSA and three CRAB isolates. The MRSA isolates were recovered from one mobile-phone sample from a professional caregiver in a surgical department and from one hand sample and one mobile-phone sample contributed by professional caregivers in internal-medicine departments. The CRAB isolates were recovered from one hand sample from a professional caregiver in a surgical department and from paired hand and mobile-phone samples from one nurse in a surgical department (**Supplementary Table 10**). No antimicrobial-resistant isolates were recovered from ICU participants.

**Table 3:** Taxon-level paired discordance between hand and mobile-phone samples. [Revised].

Taxon	Both present	Hand only	Phone only	Both absent	McNemar P	FDR q	Bonferroni P
CoNS	73	7	28	18	<0.001	0.003	0.003
*Corynebacterium* spp.	32	21	30	43	0.262	0.787	1.000
*Micrococcus* spp.	29	20	22	55	0.878	1.000	1.000
*Staphylococcus aureus*	1	4	3	118	1.000	1.000	1.000
Enterobacterales	2	1	2	121	1.000	1.000	1.000
Non-fermenting Gram-negative bacilli	1	2	1	122	1.000	1.000	1.000

Exact McNemar tests used participant-level paired presence/absence tables. The six displayed taxa formed the prespecified multiplicity family; the residual Other category was excluded. FDR q values used Benjamini–Hochberg adjustment.

## 4 Discussion

In this paired cross-sectional study of 126 physicians, nurses, and professional caregivers, hand and mobile-phone culture burdens showed a positive participant-level rank association. The association remained in the core and extended models after separate standardization of the two site-specific log-burden variables. It also remained positive after influential observations were excluded, although the smaller estimate indicates that association magnitude was partly influenced by those observations. Paired samples shared a median of one culture-defined taxon, and CoNS was the only prespecified taxon with multiplicity-adjusted discordance, with phone-only detection more frequent than hand-only detection. These findings indicate participant-level co-occurrence and partial taxonomic overlap, without establishing microbial transfer between the two sites.

Mobile-phone contamination among healthcare personnel is well documented. Systematic reviews report frequent bacterial recovery from phones across care settings but do not establish a direct causal relation with healthcare-associated infection (Olsen et al., 2020; De Groote et al., 2022; Zenbaba et al., 2023). Studies in China and other settings have also recovered skin-associated organisms and potential pathogens from phones and, in some cases, paired hand samples (Yao et al., 2022; Altunisik Toplu et al., 2026). The present study adds participant-level burden association, binary taxon overlap, and paired discordance analyses while maintaining the distinct measurement scales of hand and phone sampling.

The positive Spearman correlation is compatible with shared environmental exposure, repeated hand–phone contact, or other participant-level behaviors affecting both sites. The rank-based analysis does not require common measurement units and evaluates relative ordering across participants, while separate standardization expresses the adjusted association in standard-deviation units. These analyses do not compare the absolute contamination levels of hands and phones. The cross-sectional design also precludes temporal ordering of the association.

Taxon-level findings showed only partial overlap between paired sites. In the full sample, significant PERMANOVA and dispersion results indicated that the multivariate signal included both location and dispersion components. Neither test was significant among pairs with non-Other taxa detected at both sites, suggesting that the full-sample result was influenced by profiles with no detected taxa at one or both sites. The culture-defined categories also span different taxonomic resolutions. Genomic or other strain-level typing would be required to assess strain relatedness or source attribution.

The excess of phone-only CoNS detection may reflect repeated deposition onto a relatively stable non-living surface, differences in organism turnover between skin and phone materials, or less frequent device cleaning. Phone-cover use has been associated with mobile-phone contamination in prior observational work (Yao et al., 2022), but surface material, cleaning technique, and time since cleaning were not measured here. CoNS are common skin-associated organisms, and many are commensals, although selected species can act as opportunistic pathogens in susceptible hosts (Becker et al., 2014). The mechanism underlying the observed discordance therefore remains hypothetical and requires longitudinal sampling or intervention studies.

Occupational analyses showed that hand CFU burden differed across groups, whereas mobile-phone CFU burden did not. Variation in patient contact, caregiving tasks, hand-hygiene opportunities, or environmental exposure may contribute to these differences. Professional caregivers frequently provide bedside care and remain understudied in infection-prevention research. Because these analyses were supplementary and based on a single-center convenience sample, they should be interpreted as exploratory rather than as occupational risk estimates.

Six antimicrobial-resistant isolates were recovered from six participant-site samples contributed by five participants, comprising three MRSA and three CRAB isolates. Their distribution across departments, participant roles, and sampling sites is reported descriptively in **Supplementary Table 10**. Three of the six resistant isolates were recovered from mobile phones. Although this finding does not establish transmission, the recovery of MRSA and CRAB from personal devices used in care environments supports prospective evaluation of device-hygiene protocols. Similar resistant organisms have been recovered from healthcare workers’ phones in earlier culture-based studies (Borer et al., 2005; Akinyemi et al., 2009; Ulger et al., 2009). The small event count does not support prevalence comparisons, risk-factor modeling, or inference about transmission.

Future studies could evaluate standardized device-cleaning guidance, material compatibility, staff education, adherence, and microbiological outcomes alongside established hand-hygiene programs (Boyce and Pittet, 2002; Pittet et al., 2000; World Health Organization, 2009). Longitudinal or randomized designs are needed to determine whether these measures reduce contamination or infection.

This study has several limitations. Convenience sampling at one tertiary hospital limits generalizability, and the cross-sectional design precludes temporal or causal inference. Culture-based methods capture only culturable organisms, and the grouped taxonomic categories cannot resolve strain relatedness or source attribution; molecular typing would be required (Grice and Segre, 2011; Byrd et al., 2018; Knight et al., 2018). Hand and mobile-phone burdens used different sampling surfaces, areas, and units, so absolute burdens were not compared. Exact intervals since hand hygiene, phone use, patient contact, and phone cleaning were not recorded, and participant-level protocol-deviation data were unavailable. The full-sample PERMANOVA result was accompanied by unequal dispersion and was not retained when analysis was restricted to pairs with taxa detected at both sites. Finally, resistant isolates were uncommon and were described without inferential analysis.

## 5 Conclusion

Hand and mobile-phone culture burdens were positively associated within participants, while paired samples showed partial culture-defined taxonomic overlap. The adjusted association persisted after separate standardization and influence exclusion, although the estimate was smaller after influential observations were removed. The cross-sectional culture-based design does not establish direct microbial transmission or causality. Longitudinal paired sampling, strain-level methods, and intervention studies are needed to examine temporal relationships and coordinated hand-hygiene and mobile-phone cleaning strategies.

## Data Availability

The original contributions presented in the study are included in the article/**Supplementary Material**, further inquiries can be directed to the corresponding author/s.
