# Supplementary material for: Hand–mobile phone culture-burden associations and taxon-level concordance among healthcare-associated personnel: a paired cross-sectional study
Source: Front Cell Infect Microbiol. 2026 Aug 26;16:1929858. doi: 10.3389/fcimb.2026.1929858 (PMC13553896; doi:10.3389/fcimb.2026.1929858)
Supplement: Supplementary file 1 [file DataSheet1.docx]

Supplementary Material

# Supplementary material directory

Supplementary Table S1. Occupational distribution of CFU burden by sampling site.

Supplementary Table S2. Occupational group comparisons and Dunn-Bonferroni post-hoc results.

Supplementary Table S3. Full coefficients for standardized hand–mobile phone culture-burden regression models.

Supplementary Table S4. Secondary unstandardized log-linear regression models.

Supplementary Table S5. Regression influence diagnostics.

Supplementary Table S6. Variance inflation factors.

Supplementary Table S7. Standardized regression sensitivity analysis excluding influential observations.

Supplementary Table S8. Dominant taxa detected by sampling site.

Supplementary Table S9. Exploratory PERMANOVA and permutation-based dispersion sensitivity analyses.

Supplementary Table S10. Participant-level context for descriptively identified antimicrobial-resistant isolates.

Supplementary Figure S1. Dominant taxa by sampling site.

## Supplementary Table S1. Occupational distribution of CFU burden by sampling site.

| **Occupation** | **n** | **Hand CFU, median (IQR)** | **Mobile-phone CFU, median (IQR)** |
| --- | --- | --- | --- |
| Doctor | 41 | 21.67 (3.33-40.00) | 1300.00 (500.00-2600.00) |
| Nurse | 38 | 3.33 (0.00-16.66) | 600.00 (300.00-1425.00) |
| Professional caregiver | 47 | 25.00 (3.33-135.00) | 1100.00 (450.00-3150.00) |

Values are median (IQR). CFU, colony-forming units; IQR, interquartile range.

## Supplementary Table S2. Occupational group comparisons and Dunn-Bonferroni post-hoc results.

| **Site** | **Analysis/comparison** | **Statistic** | **Raw P** | **Adjusted P** | **Effect size or note** |
| --- | --- | --- | --- | --- | --- |
| Hand | Kruskal-Wallis by occupation | H=11.886 | 0.003 | - | epsilon-squared=0.08 |
| Mobile phone | Kruskal-Wallis by occupation | H=4.947 | 0.082 | - | epsilon-squared=0.024 |
| Hand | Doctor vs Nurse | z=2.164 | 0.030 | 0.091 | Dunn-Bonferroni post-hoc |
| Hand | Doctor vs Professional caregiver | z=-1.217 | 0.224 | 0.671 | Dunn-Bonferroni post-hoc |
| Hand | Nurse vs Professional caregiver | z=-3.426 | <0.001 | 0.002 | Dunn-Bonferroni post-hoc |
| Mobile phone | Doctor vs Nurse | z=1.988 | 0.047 | 0.14 | Dunn-Bonferroni post-hoc |
| Mobile phone | Doctor vs Professional caregiver | z=0.148 | 0.883 | 1 | Dunn-Bonferroni post-hoc |
| Mobile phone | Nurse vs Professional caregiver | z=-1.907 | 0.056 | 0.169 | Dunn-Bonferroni post-hoc |

Kruskal-Wallis tests were performed within each sampling site. Dunn post-hoc comparisons used Bonferroni adjustment.

## Supplementary Table S3. Full coefficients for standardized hand–mobile phone culture-burden regression models.

| **Model** | **Term** | **Beta (95% CI)** | **P value** |
| --- | --- | --- | --- |
| Unadjusted | Intercept | -0.000 (-0.153 to 0.153) | 1.000 |
| Unadjusted | Standardized log10(hand CFU+1) | 0.503 (0.316 to 0.691) | <0.001 |
| Core adjusted | Intercept | -0.150 (-1.399 to 1.099) | 0.814 |
| Core adjusted | Standardized log10(hand CFU+1) | 0.512 (0.291 to 0.732) | <0.001 |
| Core adjusted | Age | 0.005 (-0.020 to 0.029) | 0.700 |
| Core adjusted | Male sex | 0.209 (-0.224 to 0.642) | 0.343 |
| Core adjusted | Nurse | -0.104 (-0.535 to 0.327) | 0.637 |
| Core adjusted | Professional caregiver | -0.348 (-1.023 to 0.326) | 0.311 |
| Core adjusted | Internal medicine department | 0.099 (-0.260 to 0.457) | 0.589 |
| Core adjusted | Surgery department | 0.042 (-0.314 to 0.397) | 0.818 |
| Core adjusted | Daily handwashing frequency | 0.002 (-0.027 to 0.031) | 0.899 |
| Core adjusted | Daily alcohol handrub use | 0.004 (-0.015 to 0.024) | 0.659 |
| Core adjusted | Mobile-phone cleaning frequency | -0.047 (-0.189 to 0.095) | 0.519 |
| Extended adjusted | Intercept | -0.396 (-1.677 to 0.886) | 0.545 |
| Extended adjusted | Standardized log10(hand CFU+1) | 0.497 (0.276 to 0.718) | <0.001 |
| Extended adjusted | Age | 0.005 (-0.018 to 0.029) | 0.654 |
| Extended adjusted | Male sex | 0.222 (-0.218 to 0.662) | 0.323 |
| Extended adjusted | Nurse | -0.158 (-0.597 to 0.280) | 0.478 |
| Extended adjusted | Professional caregiver | -0.247 (-0.906 to 0.411) | 0.462 |
| Extended adjusted | Internal medicine department | 0.119 (-0.238 to 0.476) | 0.514 |
| Extended adjusted | Surgery department | 0.058 (-0.299 to 0.415) | 0.751 |
| Extended adjusted | Daily handwashing frequency | 0.002 (-0.026 to 0.031) | 0.875 |
| Extended adjusted | Daily alcohol handrub use | 0.005 (-0.015 to 0.024) | 0.637 |
| Extended adjusted | Mobile-phone cleaning frequency | -0.044 (-0.185 to 0.098) | 0.546 |
| Extended adjusted | Mobile-phone use duration | 0.039 (0.005 to 0.073) | 0.024 |

Dependent variable: standardized mobile-phone log10(CFU+1). The hand predictor was separately standardized; categorical reference groups were female sex, physician, and ICU. HC3 robust confidence intervals and P values are shown. Model fit: unadjusted model adjusted R²=0.247; core model adjusted R²=0.217; extended model adjusted R²=0.225.

## Supplementary Table S4. Secondary unstandardized log-linear regression models.

| **Model** | **Unstandardized beta (95% CI)** | **P value** | **n** | **Adjusted R²** |
| --- | --- | --- | --- | --- |
| Unadjusted | 0.548 (0.344 to 0.752) | <0.001 | 126 | 0.247 |
| Core adjusted | 0.557 (0.317 to 0.797) | <0.001 | 126 | 0.217 |
| Extended adjusted | 0.541 (0.300 to 0.782) | <0.001 | 126 | 0.225 |

These secondary models retain the original site-specific log scales. Their coefficients are not interpreted as direct comparisons of hand CFU/cm² and mobile-phone CFU/phone.

## Supplementary Table S5. Regression influence diagnostics.

| **Model** | **n** | **Parameters** | **Max leverage** | **Leverage >2p/n** | **Max Cook's D** | **Cook's D >4/n** | **\|studentized residual\| >3** | **Adjusted R^2^** |
| --- | --- | --- | --- | --- | --- | --- | --- | --- |
| Unadjusted | 126 | 2 | 0.052 | 7 | 0.093 | 11 | 1 | 0.247 |
| Core adjusted | 126 | 11 | 0.366 | 4 | 0.147 | 8 | 2 | 0.217 |
| Extended adjusted | 126 | 12 | 0.374 | 4 | 0.133 | 8 | 2 | 0.225 |

Diagnostics summarize leverage, Cook's distance, studentized residuals, and model fit.

## Supplementary Table S6. Variance inflation factors.

| **Covariate** | **Core adjusted VIF** | **Extended adjusted VIF** |
| --- | --- | --- |
| Professional caregiver | 5.827 | 6.004 |
| Age | 4.429 | 4.433 |
| Surgery department | 2.306 | 2.31 |
| Internal medicine department | 2.219 | 2.227 |
| Daily handwashing frequency | 2.188 | 2.189 |
| Nurse | 1.864 | 1.91 |
| Daily alcohol handrub use | 1.584 | 1.585 |
| Male sex | 1.495 | 1.497 |
| Mobile-phone use duration | Not included | 1.385 |
| Standardized log10(hand CFU+1) | 1.264 | 1.28 |
| Mobile-phone cleaning frequency | 1.144 | 1.145 |

VIF, variance inflation factor. The highest VIF was 6.004 for professional caregiver; VIFs for the standardized hand-burden predictor were 1.264 in the core model and 1.280 in the extended model. VIF values are unchanged by linear standardization.

## Supplementary Table S7. Standardized regression sensitivity analysis excluding influential observations.

| **Sensitivity analysis** | **n** | **Excluded observations** | **Standardized beta** | **95% CI** | **P value** | **Adjusted R²** |
| --- | --- | --- | --- | --- | --- | --- |
| Core adjusted | 126 | 0 | 0.512 | 0.291 to 0.732 | <0.001 | 0.217 |
| Core adjusted, excluding Cook's D >4/n or leverage >2p/n | 114 | 12 | 0.286 | 0.158 to 0.413 | <0.001 | 0.179 |
| Extended adjusted | 126 | 0 | 0.497 | 0.276 to 0.718 | <0.001 | 0.225 |
| Extended adjusted, excluding Cook's D >4/n or leverage >2p/n | 114 | 12 | 0.266 | 0.139 to 0.394 | <0.001 | 0.165 |

Influential observations were identified within each model using Cook's distance >4/n or leverage >2p/n. Standardized variables used full-sample means and standard deviations; HC3 robust confidence intervals are shown.

## Supplementary Table S8. Dominant taxa detected by sampling site.

| **Taxon** | **Hand records, n (%)** | **Mobile-phone records, n (%)** |
| --- | --- | --- |
| CoNS | 80 (35.2%) | 101 (38.1%) |
| *Corynebacterium spp.* | 53 (23.3%) | 62 (23.4%) |
| *Micrococcus spp.* | 49 (21.6%) | 51 (19.2%) |
| *Streptococcus spp.* | 16 (7.0%) | 18 (6.8%) |
| Bacillus and related groups | 8 (3.5%) | 4 (1.5%) |
| *Staphylococcus aureus* | 5 (2.2%) | 4 (1.5%) |
| Other Gram-negative bacilli | 4 (1.8%) | 6 (2.3%) |
| Enterobacterales | 3 (1.3%) | 4 (1.5%) |
| Molds | 3 (1.3%) | 0 (0.0%) |
| Non-fermenting Gram-negative bacilli | 3 (1.3%) | 2 (0.8%) |
| Other | 2 (0.9%) | 11 (4.2%) |
| *Enterococcus spp.* | 1 (0.4%) | 0 (0.0%) |
| Yeasts | 0 (0.0%) | 2 (0.8%) |

Taxa are summarized descriptively by detection record. Site totals were hand=227 and mobile phone=265 records. The residual Other category is reported only in this table and was excluded from Supplementary Figure S1 and all overlap, multivariate, and McNemar analyses.

## Supplementary Table S9. Exploratory PERMANOVA and permutation-based dispersion sensitivity analyses.

| **Analysis** | **Statistic** | **P value** | **Interpretation** |
| --- | --- | --- | --- |
| Full-sample paired-label PERMANOVA, Other excluded | pseudo-F=5.886 | <0.001 | Distributional difference accompanied by unequal dispersion. |
| Full-sample dispersion analysis, Other excluded | absolute difference=0.099 | <0.001 | Mean distance to centroid: hand=0.548; phone=0.449. |
| Restricted paired-label PERMANOVA, 99 both-positive pairs | pseudo-F=1.020 | 0.271 | No evidence of a site difference in the restricted subset. |
| Restricted dispersion analysis, 99 both-positive pairs | absolute difference=0.043 | 0.081 | Mean distance to centroid: hand=0.445; phone=0.402. |

Analyses used binary Bray–Curtis distances after exclusion of the residual Other category. Full-sample analyses included all 126 pairs; the distance between two all-zero profiles was set to 0. The restricted sensitivity analysis included 99 pairs with non-Other taxa detected at both sites. Each test used 9,999 within-participant label permutations. The full-sample PERMANOVA was accompanied by unequal dispersion and was not reproduced in the restricted subset.

## Supplementary Table S10. Participant-level context for descriptively identified antimicrobial-resistant isolates.

| **Department** | **Participant role** | **Sampling site** | **Organism** | **Phenotype** | **Isolates, n** | **Paired resistant detection** |
| --- | --- | --- | --- | --- | --- | --- |
| Internal medicine | Professional caregiver | Hand | *Staphylococcus aureus* | MRSA | 1 | No |
| Internal medicine | Professional caregiver | Mobile phone | *Staphylococcus aureus* | MRSA | 1 | No |
| Surgery | Professional caregiver | Mobile phone | *Staphylococcus aureus* | MRSA | 1 | No |
| Surgery | Professional caregiver | Hand | *Acinetobacter baumannii* | CRAB | 1 | No |
| Surgery | Nurse | Hand | *Acinetobacter baumannii* | CRAB | 1 | Yes; same participant |
| Surgery | Nurse | Mobile phone | *Acinetobacter baumannii* | CRAB | 1 | Yes; same participant |

MRSA, methicillin-resistant *Staphylococcus aureus*; CRAB, carbapenem-resistant *Acinetobacter baumannii*. Six antimicrobial-resistant isolates were recovered from six participant-site samples contributed by five participants (5/126); one nurse in a surgical department had CRAB recovered from both paired sites. No antimicrobial-resistant isolates were recovered from ICU participants. Strain-level relatedness between the paired CRAB isolates was not assessed. No inferential comparison was performed. Participant-level protocol-deviation data were unavailable.

## Supplementary Figure S1. Dominant taxa by sampling site.

The residual Other category was excluded from this figure, overlap metrics, multivariate analyses, and McNemar analyses; it is reported descriptively only in Supplementary Table S8.
